# Heat of Formation of *N*-Dimethylaminodiborane

**DOI:** 10.6028/jres.065A.007

**Published:** 1961-02-01

**Authors:** Walter H. Johnson, Irving Jaffe, Edward J. Prosen

## Abstract

The heat of reaction of *N*-dimethylaminodiborane with water has been determined according to the reaction:

$$\begin{array}{c} {\left({\text{CH}}_{3}\right)}_{2}{\text{NB}}_{2}H_{5}(g)+6H_{2}O(\text{liq})={\left({\text{CH}}_{3}\right)}_{2}\text{NH}(g)+2H_{3}{\text{BO}}_{3}(c)+5H_{2}(g),\\ \Delta H(25\;{}^{\circ}C)=-374.99\pm 2.71\;\text{kj}/\text{mole}\\ \;=-89.62\pm 0.65\;\text{kcal}/\text{mole}. \end{array}$$

A combination of this value with the heat of vaporization, and with the heats of formation of boric acid, dimethylamine, and water gives for liquid *N*-dimethylaminodiborane:

ΔHf°(25°C)=−36.22±0.75kcal/mole.

## 1. Introduction

The measurement of heats of reaction and formation of boron hydrides and related compounds generally presents some interesting problems. These materials, unlike compounds of carbon, hydrogen, and oxygen, do not burn completely in an oxygen bomb. A detonation usually occurs upon ignition and the combustion products include variable amounts of a residue which is insoluble even in strong acids.

In general the boron hydrides and their compounds may be thermally decomposed; this is perhaps the best method for determination of the heats of formation of boron hydrides although a rather high temperature is required. When, however, the compound contains additional elements, such as carbon and nitrogen, the reactivity of boron at elevated temperatures is likely to lead to the production of varying amounts of boron carbide and boron nitride.

A hydrolysis method, although usually longer and more involved, has one distinct advantage, in that the composition and thermodynamic state of the reaction products are usually well defined. It was for this reason that, for the present investigation, the combustion method was abandoned in favor of the hydrolysis method. The heats of reaction of *N*-dimethylaminodiborane and dimethylamine with aqueous sulfuric acid were measured; by combining these heats with appropriate auxiliary data we have obtained a value for the heat of formation of *N*-dimethylaminodiborane.

## 2. Apparatus and Materials

The calorimetric and thermometric systems and the apparatus for measurement of electrical energy have been described [1 to 4].^2^ The temperature of the calorimeter jacket was controlled at approximately 27 °C and was maintained constant within ±0.001 °C during each experiment by means of a thermostat.

The purity of the *N*-dimethylaminodiborane was determined to be 99.94 mole percent from calorimetric melting-point determination [5]. The sample was stored at −20 °C in a sealed ampoule with an internal breakoff. After each experiment, the material remaining was transferred to another similar ampoule and sealed in vacuo. By this method, all contact of the material with air or moisture was avoided and the temperature was kept at or below 0 °C.

The helium was passed through a tube containing copper oxide at 600 °C and through three absorbers containing, respectively, Ascarite, anhydrous magnesium perchlorate, and phosphorus pentoxide to remove possible traces of combustible material and water vapor.

The dimethylamine was obtained from the Matheson Company, who certified it to have a purity of not less than 98.2 percent. The purity was determined to be 98.7 mole percent from infrared spectral data,^3^ the impurities consisting chiefly of methylamine and ammonia.

The boric acid was reagent-grade material, recrystallized from water and dried in vacuo. Titration with standard alkali in the presence of d-mannitol indicated the purity to be 99.9 percent by weight.

The sulfuric acid solution was prepared by diluting concentrated, reagent-grade acid. The concentration was adjusted to 1.0 *N* by titration with standard alkali solution.

## 3. Procedure

### 3.1. Hydrolysis of *N*-Dimethylaminodiborane

The liquid *N*-dimethylaminodiborane was contained in a bubbler vessel which was immersed in an ice-water bath. The calorimetric vessel was filled with 1-*N* sulfuric acid, the total quantity of which was carefully reproduced from experiment to experiment. The calorimetric vessel and heater were then transferred to the calorimeter together with 4687 g of water, the calorimeter was assembled, and the platinum resistance thermometer was inserted.

The calorimeter was electrically preheated to the desired initial temperature (26.6 °C) and the vessel was flushed with purified helium. When thermal equilibrium was established the helium was switched to bypass the vessel. Calorimeter temperatures were observed at 2-min intervals during a 20-min initial rating period, after which helium, saturated with *N*-dimethylaminodiborane, was bubbled through the solution for 40 min. The vessel was then flushed with helium for 20 min, after which the helium was by-passed. Calorimeter temperatures were observed at 1-min intervals during the 60-min “reaction” period and at 2-min intervals during the 20-min final rating period.

The water which vaporized from the vessel during the initial flushing before the experiment, was collected in a weighed absorption-tube containing anhydrous magnesium perchlorate and phosphorus pentoxide [1]. This quantity of water represented a change in the concentration of the sulfuric acid solution in the reaction vessel, as well as a change in the energy equivalent of the calorimetric system; the corrections for these changes, however, turned out to be negligible.

The helium, the hydrogen, and the water vaporized during the experiment were passed successively through a weighed water-absorption tube, an oxidizer containing copper oxide at 600 °C, a second water-absorption tube, and a capillary flowmeter. The water vaporized from the sulfuric acid solution was collected in the first absorption-tube. The helium and hydrogen passed through the oxidizer, in which the hydrogen was burned to water, which was collected in the second absorption tube. The rate of flow of the helium was determined by means of the capillary flowmeter.

The mass of water collected in the first absorption tube served for calculation of the correction to be applied for the heat of vaporization of water from the sulfuric acid solution during the experiment. The mass of water collected in the second absorption tube was used to determine the amount of the chemical reaction.

In some cases, the resulting solutions were analyzed for boric acid and for dimethylamine. The results obtained were in substantial agreement with the stoichiometric ratios among dimethylamine, boric acid, and hydrogen.

The volume of helium was determined from rate and time observations. The helium and the *N*-dimethylaminodiborane vapor were assumed to be at room temperature when they entered the calorimeter; the helium, hydrogen, and water vapor were assumed to be at the mean temperature of the calorimeter upon leaving the system. These data were required only for the small correction for the heat capacity of the gas.

The calorimetric system was calibrated with electrical energy by use of the same general procedure except that no *N*-dimethylaminodiborane was introduced.

### 3.2. Neutralization of Dimethylamine

The experiments on the heat of neutralization of dimethylamine were performed with the same calorimetric system and experimental procedure as for *N*-dimethylaminodiborane. The gaseous dimethylamine was introduced under its own vapor pressure; helium was used for flushing the vessel before and after the experiment and also during the addition of the dimethylamine.

The quantity of reaction was determined by analysing the resulting solution for dimethylamine. The solution was placed in a distilling flask fitted with a condenser and a dropping funnel. Sodium hydroxide solution (10-*N*) was added slowly, and the dimethylamine vapors were collected in an excess of standard 0.2-*N* sulfuric acid. The flask was then slowly heated until about one-fourth of the liquid had distilled. The excess sulfuric acid was titrated using standard sodium hydroxide.

## 4. Units and Conversion Factors

The unit of energy is the joule, obtained in terms of the volt, the ohm, and the mean solar second by reference to standards maintained at NBS. For conversion to the conventional thermochemical calorie, one calorie is equivalent to 4.1840 joules.

All atomic weights were taken from the 1957 International Table of Atomic Weights [6].

## 5. Results and Calculations

The results of the calibration experiments are given in table 1. The electrical energy (*E*), corrected for the heat of vaporization of water (*q_vap_*) and for the heat carried into the system by the helium (*q_g_*), and divided by the corrected temperature rise (Δ*Rc*) [7] yields the energy equivalent of the calorimeter system (*E*_s_).

The results of the experiments on the hydrolysis of *N*-dimethylaminodiborane are given in table 2. The product of *E_s_* and Δ*Rc*, when corrected for the vaporization of water (*q_vap_*) and for the energy contributed by the helium and the dimethylamine vapor, (*q_g_*), gives the actual amount of energy evolved in the experimental process. The ratio between this amount of energy and the quantity of reaction yields the heat of reaction at the final temperature of the calorimeter (26.86 °C).

The following molar and apparent molal heat-capacities, in j/°C mole, were used for correcting the data to the process at 25 °C:
(CH_3_)_2_NB_2_H_5_(g)  Cp = 117[8],H_2_O(liq)  Cp = 75. 3[9],H_2_SO_4_(aq, 1 *N*)ΦCp = 50[10],(CH_3_)_2_NH·H_2_SO_4_ (aq, *m* = 0.04)ΦCp = 84(estimated),H_3_BO_3_(aq, *m* = 0.08)ΦCp = −82(estimated),H_2_(g)  Cp = 28.8[9],(CH_3_)_2_NH(g)  Cp = 63(estimated).The results, corrected to 25 °C, correspond to the average process:

$$\begin{array}{l} {\left({\text{CH}}_{3}\right)}_{2}{\text{NB}}_{2}H_{5}(g)+[13.75H_{2}{\text{SO}}_{4}+1506H_{2}O](\text{soln})\\ \;=[12.75H_{2}{\text{SO}}_{4}+{\left({\text{CH}}_{3}\right)}_{2}\text{NH}\cdot H_{2}{\text{SO}}_{4}+2\;H_{3}{\text{BO}}_{3}+1500\;H_{2}O](\text{soln})+5\;H_{2}(g),\\ \;\Delta H(25\;{}^{\circ}C)=-437.69\pm 2.58\;\text{kj}/\text{mole}\\ \;=-104.61\pm 0.62\;\text{kcal}/\text{mole}. \end{array}$$ (1)

The results of the experiments on the neutralization of dimethylamine in dilute sulfuric acid are given in table 3. The results, corrected to 25 °C, correspond to the average process:

$$\begin{array}{l} {\left({\text{CH}}_{3}\right)}_{2}\text{NH}(g)+[13.75\;H_{2}{\text{SO}}_{4}+1500\;H_{2}O](\text{soln})\\ \;=[12.75\;H_{2}{\text{SO}}_{4}+{\left({\text{CH}}_{3}\right)}_{2}\text{NH}\cdot H_{2}{\text{SO}}_{4}+1500\;H_{2}O](\text{soln}),\\ \;\Delta H(25\;{}^{\circ}C)=-106.04\pm 0.67\;\text{kj}/\text{mole},\\ \;=-25.34\pm 0.16\;\text{kcal}/\text{mole}. \end{array}$$ (2)

It seemed highly probable that the heat of solution of boric acid in the dilute sulfuric acid-dimethylamine sulfate solution would be very nearly the same as in pure water. This was verified when samples of boric acid, added to the dimethylamine-sulfuric acid solution, yielded results corresponding to the process:

$$\begin{array}{l} 2\;H_{3}{\text{BO}}_{3}(c)+[12.75\;H_{2}{\text{SO}}_{4}+{\left({\text{CH}}_{3}\right)}_{2}\text{NH}\cdot H_{2}{\text{SO}}_{4}+1500\;H_{2}O](\text{soln})=[12.75\;H_{2}{\text{SO}}_{4}+{\left({\text{CH}}_{3}\right)}_{2}\text{NH}\cdot H_{2}{\text{SO}}_{4}+2\;H_{3}{\text{BO}}_{3}+1500\;H_{2}O](\text{soln}),\\ \;\Delta H(25\;{}^{\circ}C)=43.34\pm 0.66\;\text{kj}/\text{mole},\\ \;=10.36\pm 0.16\;\text{kcal}/\text{mole}. \end{array}$$ (3)

The uncertainties assigned to the above values have been taken as twice the standard deviation of the mean of the experimental data combined with reasonable estimates of all other known sources of error.

The combination of eqs 1, 2, and 3 gives for the reaction of *N*-dimethylaminodiborane with water:

$$\begin{array}{l} {\left({\text{CH}}_{3}\right)}_{2}{\text{NB}}_{2}H_{5}(g)+6\;H_{2}O(\text{liq})={\left({\text{CH}}_{3}\right)}_{2}\text{NH}(g)+2\;H_{3}{\text{BO}}_{3}(c)+5\;H_{2}(g),\\ \;\Delta H(25\;{}^{\circ}C)=-374.99\pm 2.71\;\text{kj}/\text{mole},\\ \;=-89.62\pm 0.65\;\text{kcal}/\text{mole}. \end{array}$$ (4)

Jaffe [11] obtained −416.71 ± 0.10 kcal/mole for the heat of combustion of liquid dimethylamine at 25 °C. We have calculated the heat of vaporization of dimethylamine at 25 °C to be 6.03 ± 0.03 kcal/mole, from the heat capacity data of Felsing and Jessen [12] and the heat-of-vaporization data of Aston, Edinoff, and Forster [13]. The resulting heat of combustion of gaseous dimethylamine (−422.74 ± 0.11 kcal/mole), combined with the heats of formation of CO_2_(g) and H_2_O(liq) [9], yields −4.47 ± 0.12 kcal/mole for the heat of formation of gaseous dimethylamine.

Equation (4) together with the heats of formation of gaseous dimethylamine (above), liquid water [9], and crystalline boric acid (−262.16 ± 0.32 kcal/mole) [1], gives for gaseous *N*-dimethylaminodiborane: ΔHf° (25 °C) = −29.24 ± 0.80 kcal/mole.

## 6. Discussion

Before the hydrolysis method was adopted, several experiments were performed in an attempt to measure the heat of combustion of *N*-dimethylaminodiborane in an oxygen bomb. In each case a dark residue was formed which was not completely soluble in concentrated nitric acid. This residue probably consisted of a mixture of boron carbide, boron nitride, boron, and carbon. There was also uncertainty regarding the boric acid content of the liquid phase, as well as a possibility of the presence of boric oxide. Corrections were applied assuming that the deficiencies in the mass of carbon dioxide and in the amount of boric acid were due to the formation of carbon and boron. The corrected combustion process may be represented by the equation:

$$\begin{array}{l} {\left({\text{CH}}_{3}\right)}_{2}{\text{NB}}_{2}H_{5}(\text{liq})+25/4\;O_{2}(g)=2{\text{CO}}_{2}(g)+5/2\;H_{2}O(\text{liq})+2H_{3}{\text{BO}}_{3}(c)+1/2\;N_{2}(g),\\ \;\Delta H(25\;{}^{\circ}C)=-884.1\pm 4.0\;\text{kcal}/\text{mole}. \end{array}$$

The heat of formation of liquid *N*-dimethylaminodiborane, obtained from the combustion data, is found to be −39.1 ± 4.0 kcal/mole. By taking 6.89 ± 0.05 kcal/mole for the heat of vaporization we obtain −32.2 ± 4.0 kcal/mole for the heat of formation of the gas. The relatively good agreement between this value and that obtained by the hydrolysis method is believed to be fortuitous, in view of the assumptions made regarding the nature and thermodynamic states of the combustion products.

## Figures and Tables

**Table 1:** Results of the calibration experiments

Experiment No.	Δ*Rc*	*E*	*q_vap_*	*q_g_*	*E_s_*
					
	*ohm*	*j*	*j*	*j*	*j*/*ohm*
1	0.029074	6427.8	−72.3	−1.3	218553
2	.036437	8182.8	−210.6	−2.2	218734
3	.028290	6359.1	−169.4	−2.8	218696
4	.036988	8337.1	−238.3	−2.1	218901
5	.039794	9001.7	−311.9	0.4	218380
6	.036701	8163.1	−130.2	−0.7	218855
	
Mean					218686
Standard deviation of the mean					±79

**Table 2:** Results of the experiments with *N*-dimethylaminodiborane

Experiment No.	Δ*Rc*	*q_vap_*	*q_g_*	(CH_3_)_2_NB_2_H_5_	−ΔH (26.86 °C)
					
	*ohm*	*j*	*j*	*mole*	*kj*/*mole*
1	0.013645	200.0	4.8	0.007288	437.54
2	.023949	454.0	11.6	.012850	443.81
3	.009435	201.3	5.1	.005198	436.65
4	.020334	243.2	9.5	.010729	438.02
5	.023506	310.7	7.0	.012473	437.59
	
Mean					438.72
Standard deviation of the mean					±1.29

**Table 3:** Results of the experiments with dimethylamine

Experiment No.	Δ*Rc*	*q_vap_*	*q_g_*	(CH_3_)_2_NH	−ΔH (26.83 °C)
					
	*ohm*	*j*	*j*	*mole*	*kj*/*mole*
1	0.027477	100.2	7.0	0.05717	106.98
2	.028560	102.4	−2.0	.06017	105.47
3	.032783	143.4	−7.0	.06832	106.93
4	.027273	199.0	−1.2	.05816	105.95
5	.021964	87.4	−22.6	.04579	106.31
6	.016012	142.1	−21.1	.03453	104.91
	
Mean					106.09
Standard deviation of the mean					±0.33

## Footnotes

2 Figures in brackets indicate the literature references at the end of this paper

3 Obtained by J. E. Stewart, formerly of the Gas Chemistry Section of the Chemistry Division.
